# Uncovering the Regulatory Role of Proteins in EBSS-Induced Autophagy Using RNA-Seq Analysis

**DOI:** 10.3390/biology14101373

**Published:** 2025-10-08

**Authors:** Chen Ruan, Yuzhu Li, Ran Wu

**Affiliations:** 1 Henan Key Laboratory of Industrial Microbial Resources and Fermentation, Technology School of Biological and Chemical Engineering, Nanyang Institute of Technology, Nanyang 473004, China; 2 School of Life Sciences, Huazhong University of Science and Technology, Wuhan 430074, China; 3 Technology School of Chemical and Pharmaceutical Engineering, Nanyang Normal University, Nanyang 473061, China; yzllyz19@163.com; 4 Technology School of Information Engineering, Nanyang Institute of Technology, Nanyang 473004, China; wuran@nyist.edu.cn

**Keywords:** autophagy, EBSS, RNA-seq, NRK, Txnrd1

## Abstract

**Simple Summary:**

EBSS is a classical autophagy inducer that provides a special culture environment lacking amino acids and serum, causing cell starvation. However, there are almost no relevant omics data related to EBSS-induced autophagy, and consequently, autophagy-regulatory proteins have not been fully elucidated. In this study, we selected EBSS-induced autophagy as our research object and uncovered proteins that have regulatory roles in this process using RNA-seq analysis. Through the combination of omics calculation and biological experiments, this study has revealed, for the first time, that TXNRD1 has a regulatory function for autophagy activation in EBSS-induced autophagy. Our research provides useful omics information that contributes to the clarification of the autophagy mechanism.

**Abstract:**

Earle’s balanced salt solution (EBSS) is a classical autophagy inducer that provides a special culture environment lacking amino acids and serum, causing cell starvation. However, the production of relevant omics data surrounding EBSS-induced autophagy is still in the early stage. The objective of this study was to identify new potential functional proteins in the autophagy process through omics analysis. We selected EBSS-induced autophagy as our research object and uncovered autophagy-regulatory proteins using RNA-seq analysis. Western blotting showed that EBSS increased LC3B-II protein levels in NRK cells, reaching the maximum amount at 2 h of culture. Then, we used next-generation sequencing to obtain quantified RNA-seq data from cells incubated with EBSS and the bowtie–tophat–cufflinks flow path to analyze the transcriptome data. Using significant differences in the FPKM values of genes in the treated group compared with those in the control group to indicate differential expression, 470 candidate genes were selected. Subsequently, GO and KEGG analyses of these genes were performed, revealing that most of these signaling pathways were closely associated with autophagy, and to better understand the potential functions and connections of these genes, protein–protein interaction networks were studied. Considering all the conclusions of the analysis, 27 candidate genes were selected for verification, where the knockdown of *Txnrd1* decreased LC3B-II protein levels in NRK cells, consistent with the results of confocal experiments. In conclusion, we uncovered autophagy-regulatory proteins using RNA-seq analysis, with our results indicating that TXNRD1 may play a role in regulating EBSS-induced autophagy via an unknown pathway. We hope that our research can provide useful information for further autophagy omics research.

## 1. Introduction

Autophagy is a highly conserved, selective process of biodegradation in plants, animals and microbes [1], playing an important role in many physiological and pathological processes, such as nutritional deprivation, environmental stress, cancer and neurodegenerative diseases [2,3]. Many autophagy regulators have been identified as playing very important roles within the several autophagy types and signaling pathways in a multitude of studies. For example, the CDK7-CDK4 signaling axis potentiates SiNP-induced autophagy by phosphorylating RB1, thus activating E2F1 and FOXO3, with the help of autophagy-inducing nanoparticles [4,5].

A wide range of autophagy inducers have been discovered by researchers in the past. The classic autophagy inducer rapamycin inhibits the enzymatic activity of mechanistic target of rapamycin kinase (MTOR) to activate the downstream autophagy system [6]. Moreover, the class I PI3K pathway inhibitor N-acetyl-D-sphingosine, the inositol monophosphatase inhibitor carbamazepine and lithium chloride activate autophagy by inhibiting certain proteins and kinases that regulate autophagy [7]. EBSS, one of the first autophagy inducers that functions similarly to serum-free induction, provides a cell-starvation environment for the initial activation of autophagy in experiments, and scientists have found ATG proteins to have autophagy-regulatory activity in these conditions [8]; ATG16L has been found to interact with Apg5 and is closely related to the formation of autophagosomes [9]. Researchers have found numerous proteins that have regulatory functions in EBSS-induced autophagy, such as phosphatidylinositol 3-kinase, GORASP2 and p53 [10]. Until now, EBSS has primarily been used in experimental controls in the field of autophagy, with researchers using EBSS treatment as a control to ascertain whether autophagy flux is unobstructed in nanoparticle-induced autophagy [11].

In recent years, various omics technologies have been widely applied in the development of autophagy research. A multi-omics analysis study revealed the role of the autophagy-related gene AGT in chemotherapy resistance in colorectal cancer, with the gene mainly expressed in areas with malignant tumors and exhibiting distinct spatial characteristics [12]. In a recent study, protein modification omics demonstrated that LARS1 lactylation inhibits autophagy by activating mTORC1 [13]. The interaction network of the sole autophagy transmembrane protein atg9 has also been clarified with the help of the proteome and transcriptome [14]. However, there are almost no relevant omics data on the induction of autophagy by EBSS.

The objective of this study is to identify new potential functional proteins in the process of EBSS-induced autophagy through omics techniques. We selected normal rat kidney (NRK) epithelial cells as an in vitro model and applied next-generation sequencing to obtain quantified RNA-seq data on the cells when incubated with EBSS in an attempt to find new regulatory genes through bioinformatics analysis and molecular biology experimentation. We hope that our research provides useful information for autophagy omics research.

## 2. Materials and Methods

### 2.1. Reagents and Antibodies

EBSS was purchased from Procell Life Science & Technology (PB180334). Microtubule-associated protein 1 light chain 3 beta (LC3B) antibodies and β-actin (ACTB) antibodies were purchased from Proteintech Group (14600-1-AP and 18420-1-AP, Wuhan, China). Dulbecco’s modified Eagle’s medium (DMEM) and fetal bovine serum were purchased from Procell Life Science & Technology (PM150210, Wuhan, China). The small interfering (si)RNAs were purchased from RiboBio (Guangzhou, China).

### 2.2. Cell Culture

The following describes the cell culturing process: Place the NRK cells stored in the liquid nitrogen tank into a constant-temperature water bath at 37 °C. Use forceps to continuously shake the cell storage tube to facilitate the rapid thawing of the cells. After the cells are thawed, disinfect the laminar flow hood with medical alcohol, transfer the cells to a 15 mL centrifuge tube, add DEME cell culture medium containing 10% FBS and mix thoroughly. Remove the supernatant, add another 5 mL of cell culture medium, and gently vortex to ensure thorough mixing. NRK cells were incubated in DMEM supplemented with 10% fetal bovine serum, 100 U/mL penicillin and 0.1 mg/mL streptomycin. The cells were cultured in a 37 °C and 5% carbon dioxide environment.

### 2.3. Starvation Inducement of Autophagy

When the cell confluence reaches approximately 90%, the cell culture stops. At this point, we re-cultured the cells, and when the cell confluence reached 50%, performed cell starvation treatment. DEME culture fluid was removed, and the NRK cells were rinsed three times with 1× EBSS and incubated in 1× EBSS for 0, 0.5, 1 and 2 h.

### 2.4. RNA-Seq Library Preparation and Sequencing

NRK cells were treated with EBSS for 0 or 2 h, respectively, collecting >1 × 10^7^ cells at each time point and repeating the process three times. The NRK cell total RNAs were extracted by TRIeasyTM Total RNA Extraction Reagent (Shanghai Yeasen Biotechnology, Shanghai, China, 10606ES60), with the RNA concentrations from the cell samples measured with a Nanodrop 2000 spectrophotometer (Thermo Fisher Scientific, Waltham, MA, USA), the RNA quality measured with an ultraviolet–visible spectrophotometer (Merrler Toledo Company, Zurich, Switzerland, UV5) and the RNA integrity tested using an Agilent Technologies 2100 Bioanalyzer. Equal amounts of high-quality total RNA per sample were used to construct the RNA-seq libraries, with each RNA-seq library constructed with the VAHTS Stranded mRNA-seq Library Prep Kit for Illumina (Vazyme, Shanghai, China, NR602-02) and the library products sequenced on a HiSeq 4000 system (Illumina, San Diego, CA, USA). Finally, the RNA-seq raw data (ID: PRJNA1303382) were uploaded to the NCBI Sequence Read Archive (https://ncbiinsights.ncbi.nlm.nih.gov/tag/sra/, accessed on 20 August 2029).

### 2.5. Quantification and Analysis of RNA-Seq Data

For transcriptome data, the bowtie–tophat–cufflinks flow path was used for the assembly and quantification of the transcriptome data. The value of fragments per kilobase of exon model per million mapped fragments (FPKM) was recorded to estimate the gene mRNA expression levels. We then used the bioinformatics software Cuffdiff to screen for differentially expressed genes in the EBSS-induced autophagic cells. For gene ontology (GO)-based enrichment analysis, KEGG (Kyoto Encyclopedia of Genes and Genomes) enrichment analysis and heat map generation, we used the R language for data calculation and graph drawing, and for the protein–protein interaction (PPI) networks, we used the STRING database and R language for data mining. Considering the excessive number of differentially expressed genes, the samples were sorted by fold change values in descending order, selecting the top 27 candidate genes (fold change > 2.4, *p*-value < 0.05) for the subsequent experiments.

### 2.6. Immunoblotting Analysis

The protease (Roche, Basel, Switzerland, 4693116001) and phosphatase inhibitors (Roche, 4906837001) were dissolved in RIPA lysis buffer (Beyotime, Wuhan, China, P0013B), and the NRK cells were lysed with RIPA lysis buffer. Cell samples were mixed with SDS buffer and kept at 98 °C for 5 min, followed by protein separation by SDS-PAGE electrophoresis and transfer to PVFD membranes. The PVDF membranes were blocked for 2 h with 5% nonfat milk, then rinsed three times with TBST buffer. The primary and secondary antibodies were incubated with the membranes overnight at 4 °C, and the following day, the membranes were rinsed three times with TBST buffer. The Odyssey_CLx imaging system (LI-COR, Odyssey) was used to capture and analyze the immunoblotting results.

### 2.7. Immunofluorescence Microscopy Procedures

A total of 4 × 10^4^ NRK-GFP-LC3 cells were inoculated into a confocal dish for 24 h. DEME culture fluid was removed, and the NRK cells were rinsed three times with 1× EBSS before being incubated in 1× EBSS for 0, 0.5, 1 and 2 h. After the processing at each time point was complete, the cell samples were photographed using a fluorescence confocal microscope.

### 2.8. RNA Interference

Cells were transfected with siRNAs (100 nM) using Lipofectamine RNAi Max (Invitrogen, Carlsbad, CA, USA, 13778150) for 48 h according to the manufacturer’s instructions, and then treated with EBSS for 2 h. Immunoblotting analysis was performed as previously described, and the protein expression levels were quantified and analyzed using Image Studio software Ver.5.2 (LI-COR, Odyssey). Fluorescent images of cells were captured using an Olympus FV1000 (Olympus Corporation, Tokyo, Japan). Txnrd1 siRNA sense: CGGGAUAACAACAAAUGUUAUTT. Txnrd1 siRNA antisense: AUAACAUUUGUUGUUAUCCCGTT.

### 2.9. Analysis of Data

For RNA-seq analysis, the *p*-values were corrected using the FDR method.

The experimental data were analyzed by Student’s *t*-test, with * *p* < 0.05 considered a significant difference. Each sample was produced in triplicate.

## 3. Results

### 3.1. EBSS-Induced Autophagy in NRK Cells

**Figure 1:**
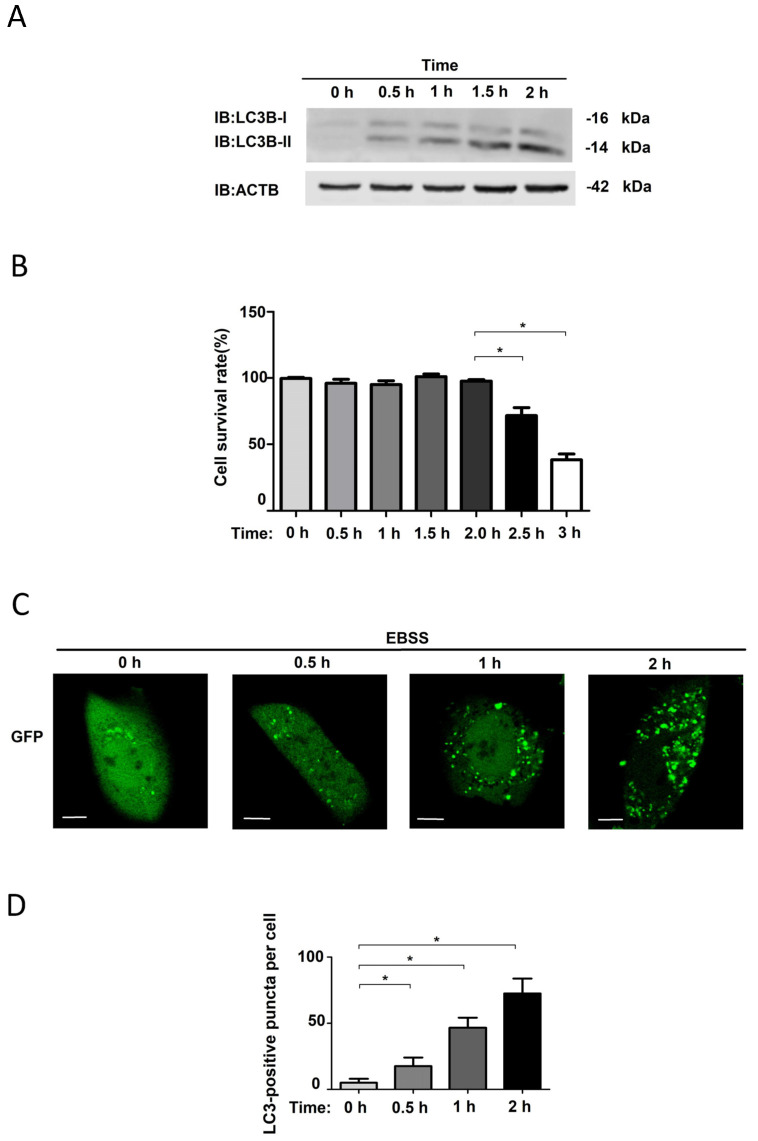
Autophagy is induced in NRK cells by EBSS. (**A**) NRK cells were incubated with EBSS for 0, 0.5, 1 and 2 h. LC3B-II protein levels were analyzed by immunoblotting. (**B**) NRK cells were incubated with EBSS, and cell survival rate was measured by the cck-8 kit. (**C**) NRK-GFP-LC3 cells were treated with EBSS for 0, 0.5, 1 and 2 h and images were captured by confocal microscope. Scale bar: 5 μm. (**D**) The average number of GFP-LC3 puncta per cell was counted in (**C**) at 0, 0.5, 1 and 2 h, respectively. GFP-LC3 puncta were quantified in ≥30 cells. *, *p* < 0.05.

To detect the effect of autophagy induction by EBSS on NRK cells, the cells were treated with EBSS for 0 h, 0.5 h, 1 h and 2 h. Western blotting showed that the protein levels of LC3B-II increased with time and reached their maximum at 2 h (Figure 1A).Cell survival rates were also detected (Figure 1B), and we thus further investigated the numbers of GFP-LC3 puncta in EBSS-induced autophagic NRK-GFP-LC3 cells [14,15,16,17]. With the help of fluorescence microscopy, we found that NRK-GFP-LC3 cells treated with EBSS had increased numbers of GFP-LC3 puncta, and longer treatments had stronger inductive effects, with the maximum number reached at 2 h (Figure 1C,D). Taken together, these results demonstrated that EBSS could activate autophagy in NRK cells and that the LC3B-II protein and GFP-LC3 puncta levels reached their maximum after 2 h.

### 3.2. Quantification and Analysis of RNA-Seq Data for EBSS-Induced Autophagic NRK Cells

**Figure 2:**
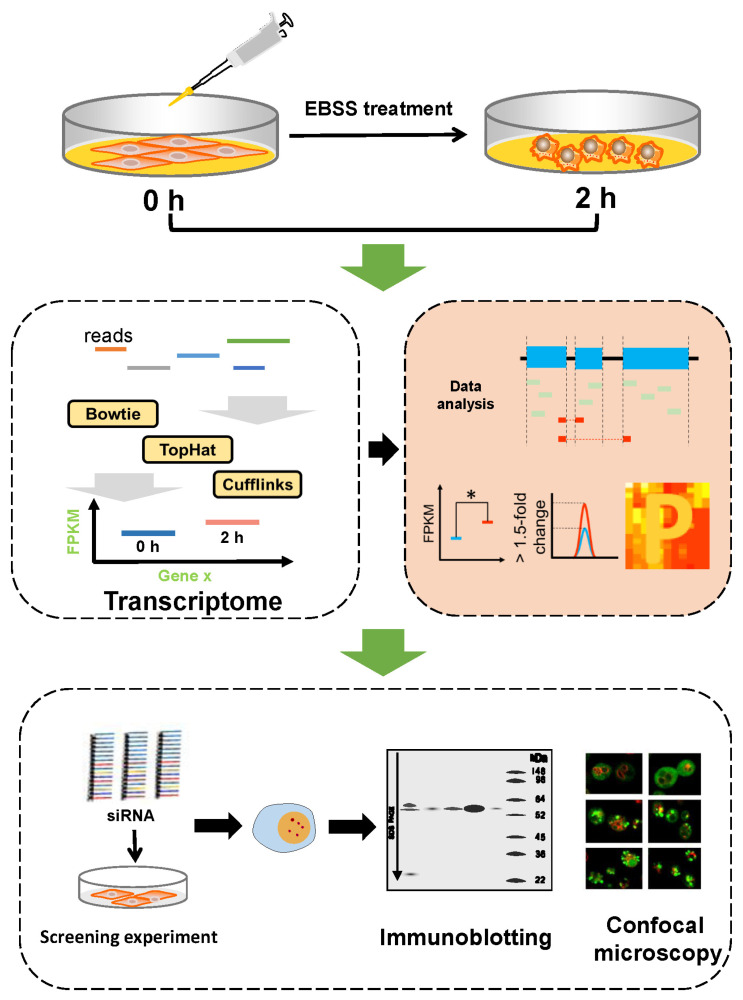
Experimental procedure of the study. The NRK cells were incubated for 0 and 2 h, and we then used next-generation sequencing technology to quantify the cell sample RNA-seq data. The bowtie–tophat–cufflinks flow path was used to analyze the transcriptome data. We used the bioinformatics software Cuffdiff to screen the differentially expressed genes in EBSS-induced autophagy. If the FPKM value of the treated group was significantly different from that of the control group, the gene was considered differential. Finally, we used gene knockdown technology, Western blotting and confocal microscopy to verify the prediction results. *, *p* < 0.05.

From the above results, we found that 2 h of EBSS treatment efficiently induced autophagy in NRK cells. In this work, we hypothesized that there would be noticeable changes to the omics levels of proteins with regulatory functions in EBSS-induced autophagy when cells were treated with EBSS. To fully excavate the functional proteins and probe the molecular changes during EBSS-induced autophagy, we used EBSS to treat NRK cells for 0 h and 2 h and applied next-generation sequencing to quantify the transcriptome data and collect gene expression levels (Figure 2).

**Figure 3:**
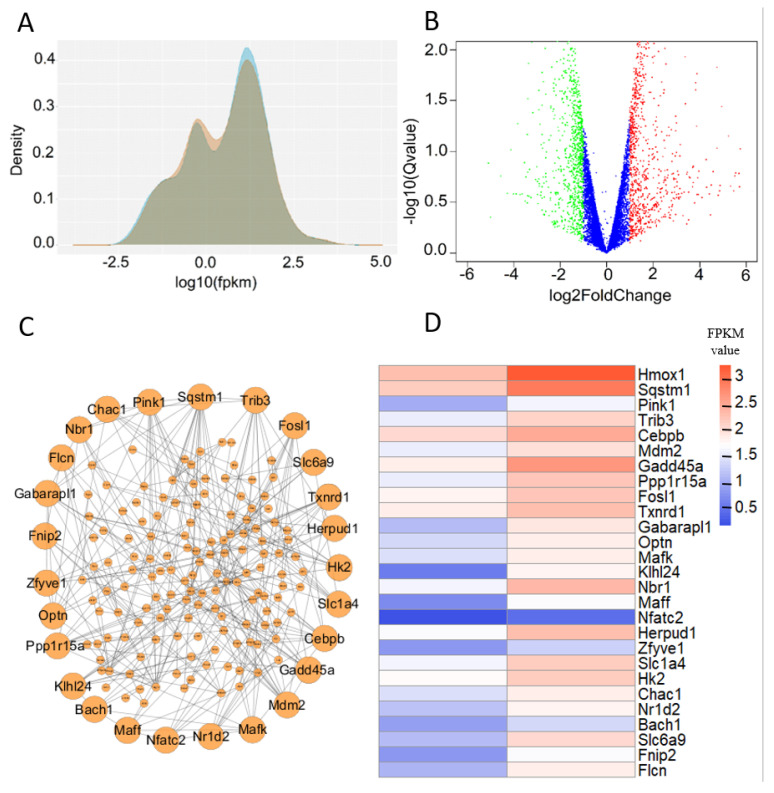
Quantitative analysis of transcriptome data. (**A**) The density distribution of all the genes in the transcriptome data. (**B**) Upregulated and downregulated genes. (**C**) Identified PPI networks. (**D**) The FPKM values of the 27 candidate genes at 0 h and 2 h in EBSS-induced autophagy.

In our data, there were 11,852 genes mapped with at least one read (Table S1). From Figure 3A, it can be seen from the omics data that the density distribution of the genes was even, which reflects the absence of errors in the preparation and sequencing of the transcriptome samples. Volcano plots are a type of scatter plot that combine measures of statistical significance (such as *p*-value) with the amplitude of variation in statistical tests, used here to quickly and intuitively identify datapoints that are significantly different. In Figure 3B, 509 genes are shown to be upregulated and 569 genes downregulated in cells treated for 2 h (|log2(fold_change)| > 1). Comparing gene transcription levels between the 2 h treatment group and 0 h treatment group, if the FPKM value of a gene in the treated group was significantly different from that of the control group (FPKM > 1, log2(fold_change) > 1, *p*-value < 0.05), it was considered differential. We found 470 genes that may have regulatory function (Table S2).

**Figure 4:**
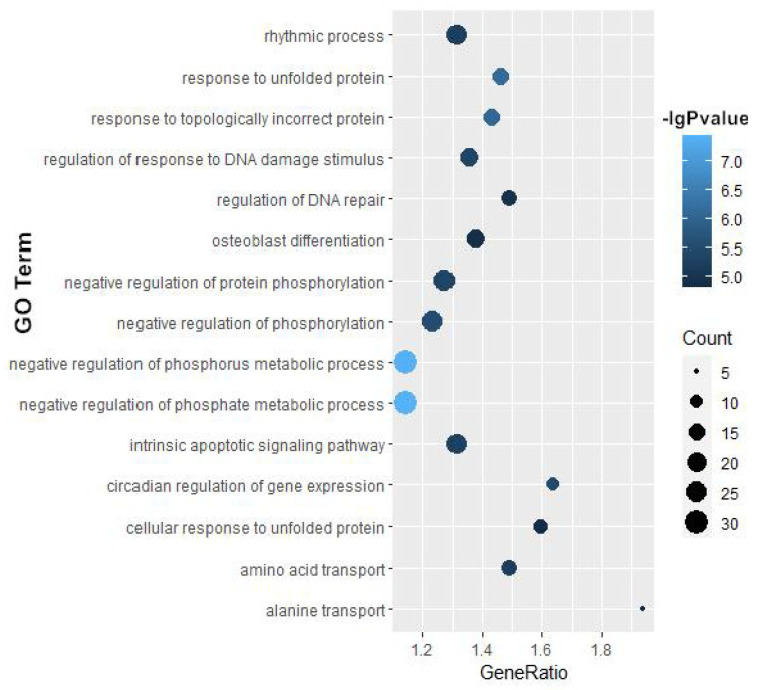
The GO-based enrichment results of biological processes.

**Figure 5:**
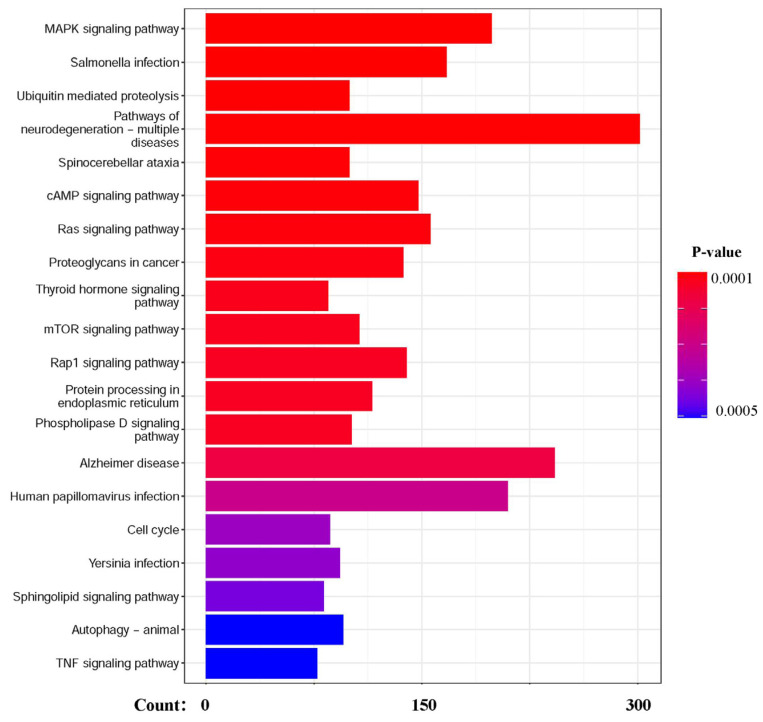
The KEGG-based enrichment results of biological processes.

Next, for a better understanding of the regulatory roles of these 470 genes, GO and KEGG enrichment analysis were performed, and numerous significantly enriched biological pathways were detected (*p*-value < 10^−3^). Interestingly, we found that most of these signaling pathways were validated to be closely associated with autophagy (Figure 4 and Figure 5), such as the responses to unfolded protein (GO:0006986) and topologically incorrect protein (GO:0035966), which have been reported to be crucial in autophagy. Moreover, responses to both oxygen levels (GO:0070482) and amino acid transport (GO:0006865) are known to participate in autophagy regulation.

Next, we explored the relationships between these proteins, making PPI networks, with the proteins with the most interaction effects shown in Figure 3C. Considering all the data analysis results, we choose the top 27 candidate genes (fold change > 2.4, *p*-value < 0.05) and examined their regulatory function in EBSS-induced autophagy (Figure 3D).

### 3.3. Validation of Regulatory Function of Candidate Proteins

**Figure 6:**
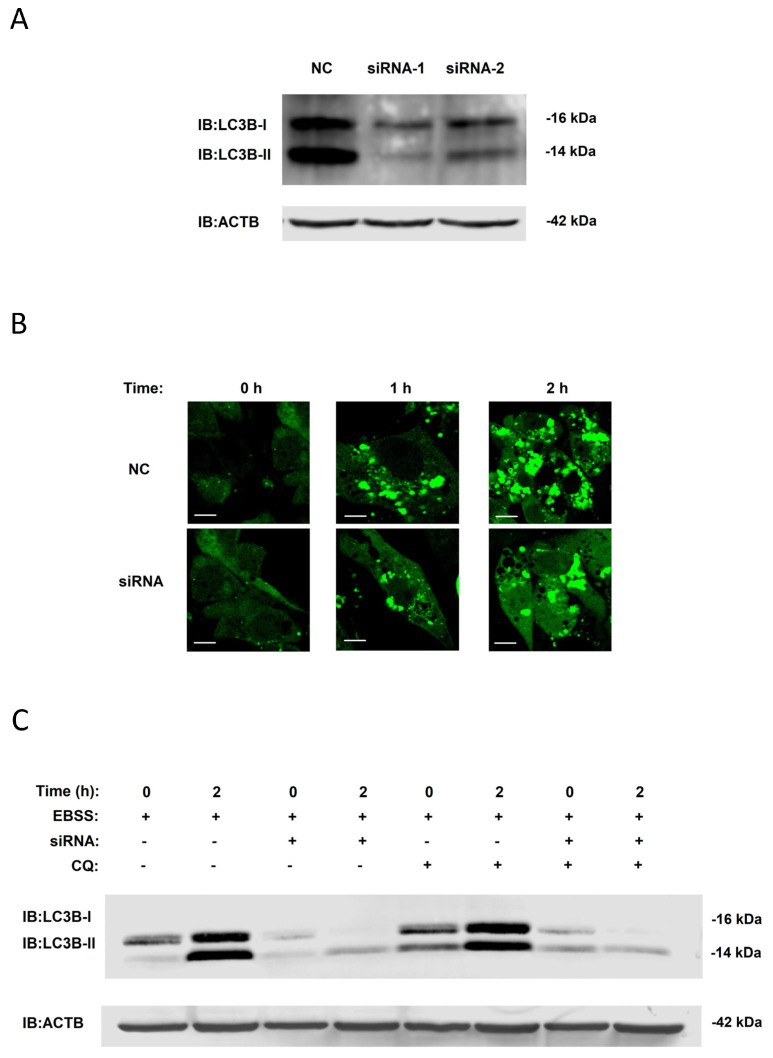
Validation of the regulatory function of proteins. (**A**) To further confirm the function of *Txnrd1* in EBSS-induced autophagy, siRNA-mediated knockdown experiments were performed of LC3-II and ACTB protein levels were measured by Western blot. (**B**) NRK-GFP-LC3 cells were individually transfected with specific siRNAs for 48 h and then treated with the EBSS for 0, 1 and 2 h. DMSO was a negative control group. The images were captured by a confocal microscope. Scale bar: 5 μm. (**C**) NRK cells were incubated with siRNAs for 48 h and treated with CQ for 8 h followed by EBSS for 2 h. The LC3-II and ACTB protein levels were then measured by Western blot.

With the help of next-generation sequencing technology and bioinformatics analysis, we choose 27 candidate genes and analyzed their function in EBSS-induced autophagy, further verifying our results using a small interfering RNA (siRNA) library designed with siRNA duplexes for each protein. The NRK cells were individually transfected with an siRNA for 48 h and then incubated with EBSS for 2 h before we collected the cell samples and detected LC3B-II protein levels by Western blot assay. Here, we also found a new gene, *Txnrd1*, that appeared to influence LC3B-II protein expression. To further confirm the function of TXNRD1 in EBSS-induced autophagy, we completed two additional siRNA-mediated knockdown experiments (Figure 6A), further investigating the numbers of GFP-LC3 puncta in NRK-GFP-LC3 cells by fluorescence microscopy and finding that *Txnrd1* knockdown reduced GFP-LC3 puncta numbers after 1 h and 2 h of culture in EBSS (Figure 6B), consistent with our knockdown experiments (Figure 6A).

From the above results, we know that *Txnrd1* knockdown causes a decrease in LC3B-II expression. There may be two reasons for this decline: one is the inhibition of the activation of upstream autophagy, and the other is an improvement in downstream autophagy degradation. To address these issues, Chloroquine (CQ), an autophagy inhibitor, was added to the knockdown experimental system of EBSS-induced autophagy, and we found that its addition did not affect the significant decrease in intracellular LC3-II protein content (Figure 6C). It can be concluded that TXNRD1 plays a role in the autophagy activation process, via an unknown pathway, but not in the later stages of autophagy.

## 4. Discussion

EBSS has been used in many autophagy research projects, almost always as a positive control, but the details of the relationship between EBSS and autophagy are still not clear. In this study, we selected NRK cells as the study model, as they have low initial autophagy levels. One limitation of this study is that its central conclusions are mainly based on a single NRK cell line, and no confirmatory experiments on other cell lines were conducted. In future in-depth research, we will conduct more thorough verification.

We detected LC3B-II protein levels when the NRK cells were incubated with EBSS for 1 h and 2 h (Figure 1A) and found that these LC3B-II levels continuously increased over time, indicating that, during the formation of the autophagosome membrane, the LC3B precursor is being cleaved by ATG4, and the autophagosome is gradually forming; with the help of confocal microscopy, we also found that autophagy intensity reached its maximum at 2 h (Figure 1C,D). These two experimental results are mutually corroborative. Our results indicate that EBSS activated autophagy in NRK cells. Previous studies have shown similar results, with one study pointing out that EBSS starvation treatment could significantly increase the autophagosome numbers in nasopharyngeal carcinoma cells [18]. In the quest to explore the relationship between the liver and obesity, researchers have also found that EBSS could activate autophagy in mice liver cells [19].

As shown in Figure 1B, we found that after the cells were treated for 2 h, their survival rates significantly decreased, indicating that, after 2 h of EBSS treatment, autophagy reached its peak and autophagic death eventually occurred. In other studies, EBSS starvation treatment induced autophagy in pig fibroblast cells, with LC3B-II protein levels significantly increasing after 4 h [8]. In HeLa cells, autophagosome and autolysosome levels significantly increased when cells were incubated in EBSS medium for 1 h, with extremely significant increases at 4 h [20,21]. These different autophagy induction times in HBSS cells may be closely related to specific cell types.

Next-generation sequencing was used to obtain quantitative RNA-seq data for the cell samples incubated with EBSS at two time points (0 h and 2 h), a design derived from the results in Figure 1B, selecting the time point at which the autophagy effect was the strongest for analysis. However, this binary design may overlook dynamic transcriptional changes occurring earlier or later, resulting in the loss of information. Including an intermediate (e.g., 1 h) or longer (4 h) time point would be better, providing a more comprehensive picture.

With the help of RNA interference and Western blot assays, we found that the expression of a new gene, Txnrd1, influenced LC3B-II levels (Figure 6A), suggesting that the Txnrd1 functions to affect autophagy in cells. Not only that, but during the verification process, we also found several genes which have been reported as having regulatory functions and mechanisms during autophagy, including Gabarapl1 and Sqstm1. SQSTM1 is an important marker protein in autophagy, playing several roles in autophagy regulation [11], with researchers finding that reducing SQSTM1 expression could significantly affect autophagy activation in cancer cells [22]. In addition, the GABARAP family is essential for phagophore initiation and autophagosome maturation [23], and the FLCN-GABARAP signaling pathway is associated with FNIP2, regulated by ULK1 [24]. In the face of this evidence, classical experimental methods can still uncover new clarifying information.

With the help of a confocal microscope, We observed that Txnrd1 expression was correlated with the number of GFP-LC3 puncta (Figure 6B), with mutually confirmatory experimental data. In the CQ experiment, we found that the addition of the autophagy inhibitor CQ did not affect the significant decrease in intracellular LC3-II protein content. Therefore, it can be concluded that Txnrd1 plays a role in autophagy activation, via an unknown pathway, but not in the later stages of autophagy. Coincidentally, scientists have also demonstrated that Txnrd1 plays a key role in ROS-mediated autophagy [25], a conclusion which supports our results.

TXNRD1 is one of the members of the selenoprotein family, with selenocysteine (Sec) as its common catalytic activity center, which comprises the main carriers of selenium for physiological functions [26]. Selenoproteins exist primarily in the form of redox enzymes and carry out very diverse functions integrally related to the development of diverse tumors, such as the regulation of cellular oxidative stress, immune function, ER stress, and autophagy [27,28]. In rat ovaries, rapamycin treatment has been shown to lead to an increase in transcriptional TXNRD1 level [29], and more importantly, one report clearly suggested the key role and potential interactions of the selenoproteins Txnrd1, Txnrd3, Selp, GPX2, Dio3 and Selr in ROS-mediated autophagy [25]. The mechanisms relating TXNRD1 to autophagy remain unclear, but according to the available literature, the selenoprotein family has complex interactions and regulatory relationships with cellular autophagy.

## 5. Conclusions

EBSS was one of the first autophagy inducers to be used experimentally, but there are almost no relevant omics data that reveal its autophagy-inducing mechanisms. In this study, a transcriptome data approach uncovered the regulatory roles of proteins in EBSS-induced autophagy, indicating that TXNRD1 plays a role in regulating EBSS-induced autophagy via an unknown pathway. However, the related regulation mechanisms of this protein need to be studied further. We hope that our bioinformatics analysis of transcriptome data will be helpful for those wishing to further elucidate the autophagy mechanisms of EBSS.

## Data Availability

The original data presented in the study are openly available in the NCBI SRA database at https://dataview.ncbi.nlm.nih.gov/object/PRJNA1303382?reviewer=ml4bk1q4sjj8m70v06n19o0dto, accessed on 20 August 2029.

## Supplementary Materials

The following supporting information can be downloaded at: https://www.mdpi.com/article/10.3390/biology14101373/s1. Table S1: An overview of data of each gene for RNA-seq; Table S2: The detailed results of 470 genes.

## Abbreviations

The following abbreviations are used in this manuscript:

EBSS	Earle’s balanced salt solution
MTOR	mechanistic target of rapamycin kinase
NRK	normal rat kidney
LC3B	microtubule-associated protein 1 light chain 3 beta
ACTB	β-actin
FPKM	value of fragments per kilobase of exon model per million mapped fragments
GO	gene ontology
KEGG	Kyoto Encyclopedia of Genes and Genomes
PPI	protein–protein interaction
siRNA	small interfering RNA
