# Supplementary figures and images for: Uncovering the Regulatory Role of Proteins in EBSS-Induced Autophagy Using RNA-Seq Analysis
Source: Biology (Basel). 2025 Oct 8;14(10):1373. doi: 10.3390/biology14101373 (PMC12561780; doi:10.3390/biology14101373)

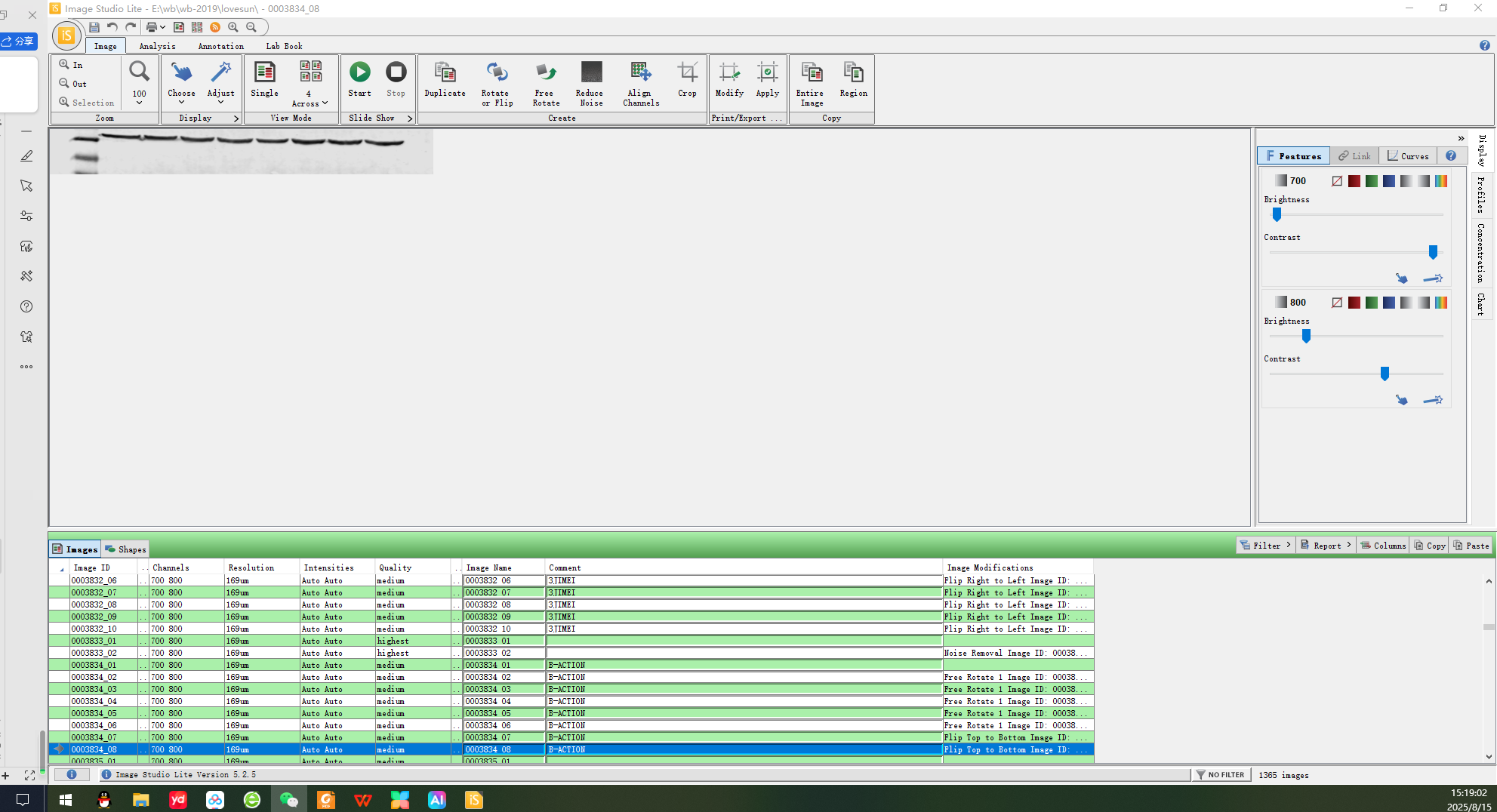

Supplement: Supplementary file 1 [file biology-14-01373-s001.zip › figure1 A-ACTB.png]

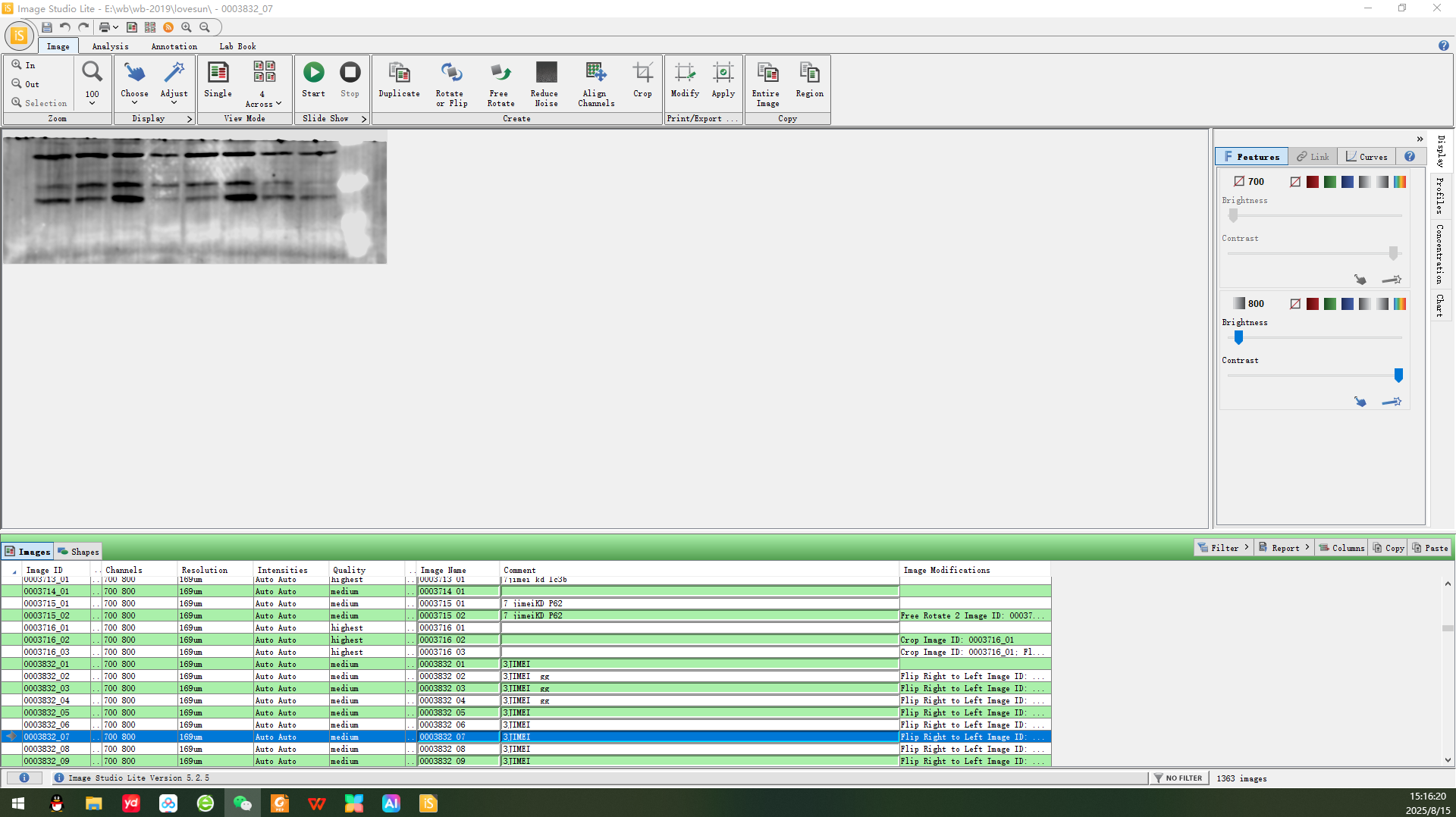

Supplement: Supplementary file 1 [file biology-14-01373-s001.zip › figure1 A-LC3B.png]

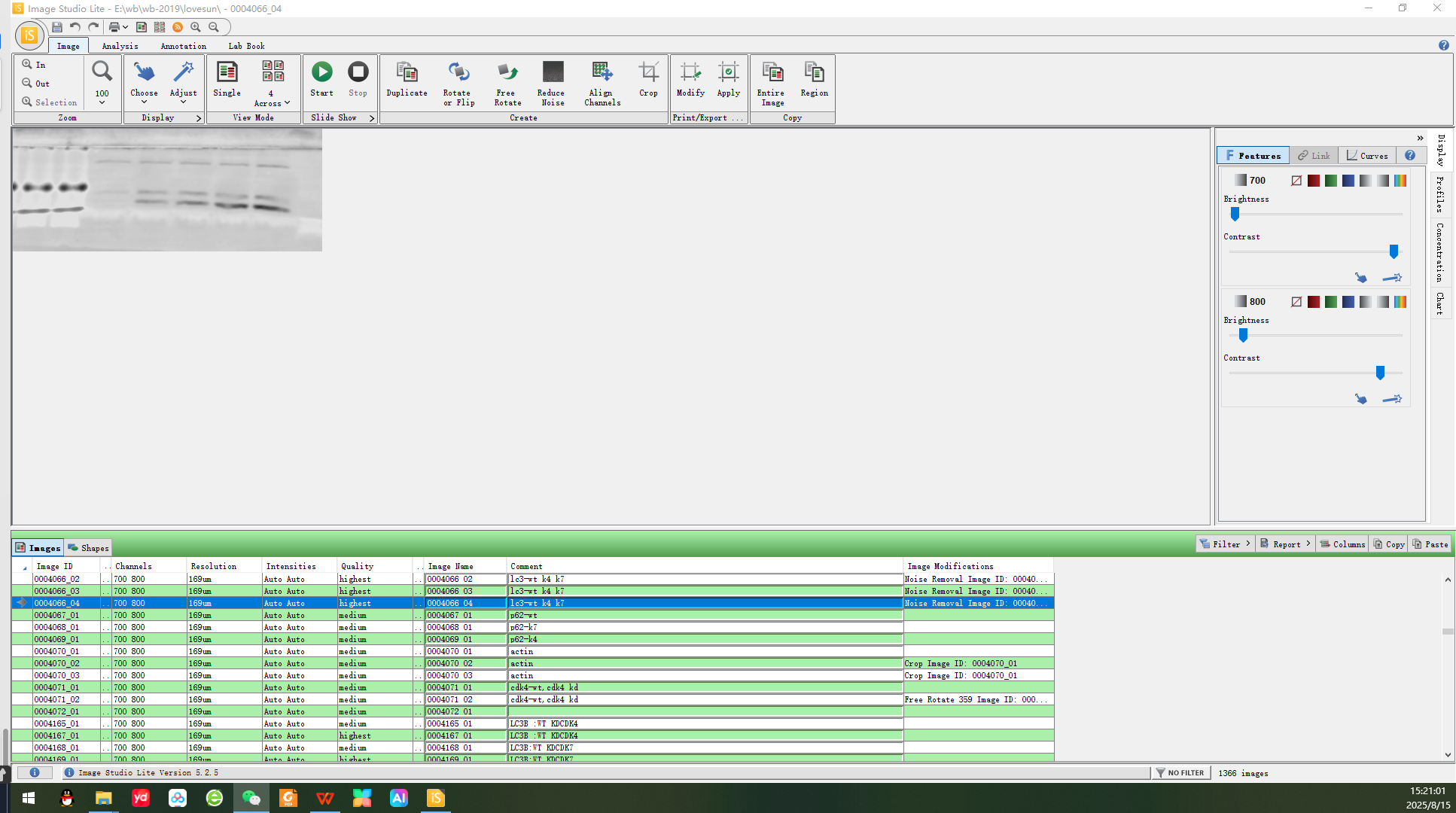

Supplement: Supplementary file 1 [file biology-14-01373-s001.zip › figure4 A-LC3B.png]
